# Assessing the model transferability for prediction of transcription factor binding sites based on chromatin accessibility

**DOI:** 10.1186/s12859-017-1769-7

**Published:** 2017-07-27

**Authors:** Sheng Liu, Cristina Zibetti, Jun Wan, Guohua Wang, Seth Blackshaw, Jiang Qian

**Affiliations:** 1 Department of Ophthalmology, Johns Hopkins University School of Medicine, Baltimore, 21287 MD USA; 2 Solomon H. Snyder Department of Neuroscience, Johns Hopkins University School of Medicine, Baltimore, 21287 MD USA; 3 Department of Neurology, Johns Hopkins University School of Medicine, Baltimore, 21287 MD USA; 4 Centre for Human Systems Biology, Johns Hopkins University School of Medicine, Baltimore, 21287 MD USA; 5 Institute for Cell Engineering, Johns Hopkins University School of Medicine, Baltimore, 21287 MD USA

**Keywords:** Transcription factor binding prediction, Chromatin accessibility, Machine learning, Feature selection

## Abstract

**Background:**

Computational prediction of transcription factor (TF) binding sites in different cell types is challenging. Recent technology development allows us to determine the genome-wide chromatin accessibility in various cellular and developmental contexts. The chromatin accessibility profiles provide useful information in prediction of TF binding events in various physiological conditions. Furthermore, ChIP-Seq analysis was used to determine genome-wide binding sites for a range of different TFs in multiple cell types. Integration of these two types of genomic information can improve the prediction of TF binding events.

**Results:**

We assessed to what extent a model built upon on other TFs and/or other cell types could be used to predict the binding sites of TFs of interest. A random forest model was built using a set of cell type-independent features such as specific sequences recognized by the TFs and evolutionary conservation, as well as cell type-specific features derived from chromatin accessibility data. Our analysis suggested that the models learned from other TFs and/or cell lines performed almost as well as the model learned from the target TF in the cell type of interest. Interestingly, models based on multiple TFs performed better than single-TF models. Finally, we proposed a universal model, BPAC, which was generated using ChIP-Seq data from multiple TFs in various cell types.

**Conclusion:**

Integrating chromatin accessibility information with sequence information improves prediction of TF binding.The prediction of TF binding is transferable across TFs and/or cell lines suggesting there are a set of universal “rules”. A computational tool was developed to predict TF binding sites based on the universal “rules”.

**Electronic supplementary material:**

The online version of this article (doi:10.1186/s12859-017-1769-7) contains supplementary material, which is available to authorized users.

## Background

Transcription factors (TFs) bind to specific DNA sequences and regulate expression of downstream genes. Prediction of TF binding sites in a particular cell type is still a considerable challenge, because the predictions simply based on TF binding consensus sequences often generate a large number of false positives. A number of computational approaches have been proposed to improve the prediction of TF binding sites (TFBS) [1, 2]. For example, integration of multiple lines of evidences, including sequence conservation, binding site conservation, gene ontology functional annotation and location relative to transcription start sites can improve the prediction of TF binding sites [3–5]. Other groups used DNA 3D structural information to model TF binding specificities [6–8]. A few groups showed that context specific TF bindings correlate with specific co-occurring sequence motifs and evolutional conservation [9–12]. Some groups attempted to use more accurate description of TF binding sites such as within-motif dependence [13]. A recent paper presented [14] a model that predicts TF binding well based on a small fraction of information across TF and cell lines from available ChIP-seq data. All these methods of analyzing TF binding utilized static genomic features that do not reflect the highly tissue- and/or cell-specific properties of actual TFBS.

Since most of TFs only bind to chromatin accessible regions, integration of chromatin accessibility datasets can greatly help improve the TF binding site prediction. First, regions of open chromatin comprise only 2.8–3.2% of genome, which reduces the prediction space and potential false positives. Second, differences in chromatin accessibility are cell type specific, and integration of the information will reflect the dynamic nature of TFBS in different cell types. Chromatin accessibility can be determined by DNase-Seq [15–17] or ATAC-Seq [18, 19], and many of these datasets have become available in diverse cell and tissue types.

Different types of computational approaches have been developed to utilize chromatin accessibility information for TFBS prediction. One important approach is to identify footprints of TFBS from DNase-Seq or ATAC-Seq profiles directly. Since proteins protect the bound DNA sequences from cutting by DNase I, the cut frequency is much lower at TFBS, resulting in a footprint in DNase-Seq profiles. The DNA sequences located in the footprints can be then used to predict the TFs that bind to the footprint sequences. Several programs have been developed to predict the TFBS based on footprints, including HINT, DNase2TF and PIQ [20–26]. Due to the intrinsic sequence bias of DNase and short residence time of some TFs [24, 26–28], for some TFs, it is hard to predict their binding from DNase/ATAC data even after bias was corrected [27]. Other approaches do not explicitly pinpoint the location of the footprint [27, 29–33]. For example, a statistical approach was developed to distinguish the DNA sequences actually bound by TFs. The approach, CENTIPEDE, utilized a hierarchical Bayesian mixture model to infer TF binding sites, making the assumption that the DNase-Seq profile surrounding the TFBS are different from those not surrounding the TFBS [29]. This approach integrates features such as position weight matrix (PWM) score, conservation score, distance to transcription start sites (TSS), and cut counts in 200 bp window around the site. Similarly, epigenetic profiles generated from DNase-Seq and other epigenetic modification data, H3K4me1, H3K4me3, H3K9Ac, and H3K27Ac ChIP-Seq data were incorporated to predict active TF binding based on standard motif model [30]. Yet another tactic was taken by utilizing ChIP-Seq to generate a discriminative flexible k-mers support vector machine (SVM) model, and used this to generate a discriminative spatial DNase SVM model using DNase read counts located around ChIP-Seq peak regions [31]. BinDNase binned the candidate binding sites and their flanking regions. The feature sets were then generated by different ways of merging the bins. Cut profiles of each merged bin for the feature set were used to train and predict binding using logistic regression. The feature set with best prediction is chosen [33]. In another study, TF binding site occupancy was predicted using a selection of sequence intrinsic and cell-type specific chromatin features in [34]. Most of these approaches are geared toward a specific TF and are unsupervised.

In this work, we attempted to extract features from chromatin accessibility data and build models based on existing TF ChIP-seq data. For the supervised learning model, it is an important issue which datasets are chosen to build the models. However, it has not been systematically assessed to what extent the models learned from other TFs in other cell types can be used to predict TF binding events. For this purpose, we first develop a new algorithm using a set of genomic features to predict TF binding sites. We then extensively evaluated the transferability using this algorithm, and found that the model learned from multiple TFs performed well to predict the binding sites for other TFs in other cell types. A general model, referred as TF Binding Prediction from accessibility data (BPAC), is thus built to predict TFs in a cell type, if the chromatin accessibility data (DNase-Seq or ATAC-Seq data) are available for the cell type. We also make available a web server and software package for users.

## Methods

### Candidate TF binding site identification

Transcription factor binding motifs were obtained from TRANSFAC [35]. TF motifs used in this study are listed in Additional file 1: Table S1. TRANSFAC matrices were converted to log-odd matrix format of the motifs using trasfac2meme [36]. FIMO [37] was used to scan the genome for candidate binding sites. The PWM score of each genomic position was computed by summing the appropriate entries from each column of the PWM that represents the TF motif, which is used as a feature. We used 1e-4 as cutoff as a match to the PWM. Only the matched positions will be considered for further analysis. We then predicted the actual binding sites among these candidate binding sites based on the motif search.

### ChIP-Seq, DNase-Seq, and ATAC-Seq data processing

Uniformly processed peaks from ChIP-Seq were obtained from the ENCODE [38] section of UCSC Genome Browser (http://genome.ucsc.edu/ENCODE/, [39, 40]). The February 2009 human genome (NCBI Build 37, hg19 assembly) was used as a reference genome. DNase-Seq alignment files were retrieved from the ENCODE. Dnase-Seq and ChIP-Seq used in this study are listed in Additional file 1: Table S2. Read profiles were generated from sequencing reads piled up at each base of the genome. The cut profiles were generated from the two nucleotides at each end of a read. Read profiles and cut profiles were extracted from the alignment files using a customized Python script based on pyDNase [23].

### Features used in the model

Features used in this study are shown in Table 1. PWM scores were scores from FIMO scan of the genome with uniform background letter frequencies. The candidate sites were first determined by scanning the PWM of each TF in human genome. Conservation scores were based on 100 way phastCons scores, which were retrieved from UCSC Genome Browser [39, 41]. Distance to TSS was calculated using BedTools [42] closest command, and the TSS definition was obtained from UCSC table browser choosing Ensembl model. Both protein-coding and non-coding RNAs were considered in the study. Read profiles and cut profiles at each base were generated from bam file and converted to bigWig format using wiggleToBigWig [43]. The average read and cut profile over all bases at each candidate binding site were then extracted. We use the same length as the length of binding site for upstream or downstream measurement. The average read and cut profiles at all bases upstream or downstream of each candidate binding site were extracted from bigWig files. The footprint score, *fp*, was calculated as:

1 $$\begin{array}{@{}rcl@{}}  {{fp}} \,=\,\! \frac{{average\ counts\ upstream + average\ counts\ downstream + pseudocount}}{{average\ counts\ at\ binding\ site + pseudocount}}, \end{array} $$

**Table 1:** Features used in the prediction

Features	Description
PWM score	The score DNA sequence against position weight matrix
Conservation score	PhastCons conservation score for multiple alignments of 99 vertebrate genomes to the human genome
Distance to TSS	Distance to transcription start site
Reads at site	Average reads at the binding site
Cut counts at site	Average cut counts at the binding site
Upstream reads	Average reads upstream of the binding site
Downstream reads	Average reads downstream of the binding site
Upstream cut counts	Average cut counts upstream of the binding site
Downstream cut counts	Average cut counts downstream of the binding Site
Reads footprint score	Average footprint score based on reads profile
Cut counts footprint score	Average footprint score based on cut profile

Where necessary, pseudo-count is added to avoid division by zero and is set to one in this study. The higher the value of *fp*, the stronger the footprint.

### Model construction

A prediction model was constructed by random forest classification algorithm [44, 45], which was obtained from scikit-learn package. In a random forest, an ensemble of decision trees is generated from randomly chosen subset of samples and features. The final prediction is an average of votes of all decision trees. Random forests can handle mixed type of data, require less pre-processing of data, and is one of the state of the art machine learning algorithm, making it suitable for evaluation for transferability in one setting. The number of decision trees was set to 100. Since we were interested in the transferability of the models, we chose the same number of trees for each model. Indeed, out of bag error rate analysis demonstrated that the number of trees of 100 was in error rate stable region. The size of subset of features was set to nearest integer of square root of number of all features. The model predicts whether a candidate binding site is an actual binding site. Different sets of features illustrated in the previous section were used to test the performance of the resulting model with the selected set of features.

### Prediction evaluation

ChIP-Seq was used to evaluate the performance after prediction was made on test set. If a TFBS site overlaps with a ChIP-Seq peak, it is considered as actual binding, i.e., bound, otherwise, it is unbound. Bound binding sites form a positive set, while unbound binding sites form negative set. We mainly used area under receiver operation characteristic curve (AUC) to access the performance as well as area under precision recall curve (AUPR). Given a binary classifier, there are four possible outcomes comparing prediction with ground truth: prediction as positive that is actually positive, which is called true positives (TP), prediction is negative that is actually negative, which is called true negatives (TN), prediction is positive but is actually negative, which is called false positives (FP), and prediction is negative but actually is positive, which is called false negatives (FN). The ratio of true positives over the sum of ground truth positives is called true positive rate (TPR or recall), i.e.: TPR = TP / (TP+FN). The ratio of false positive over the sum of ground truth negatives is called false positive rate (FPR), i.e.: FPR = FP / (FP+TN). Receiver operating characteristic (ROC) curve is constructed by plotting TPR against FPR at different thresholds. AUC measures the aggregated classification performance. The higher the better performance is assumed. Specificity or true negative rate is the ratio of true negatives over the ground truth negative. It is 1-FPR. Precision is the ratio of true positives over the sum of predicted positives, i.e.: precision = TP / (TP+FP). The overall performance of precision and recall can be represented by the precision recall curve. AUPR summarizes the classification performance in terms of precision and recall.

### Performance of CENTIPEDE, HINT-BC, and DNASE2TF

We ran the methods using default settings. Identified binding sites (CENTIPEDE, HINT-BC) or footprint (DNASE2TF) were matched with candidate binding sites scanned by FIMO. The matched binding sites are considered as positive prediction for each method. When calculating AUC, only those candidate binding sites scanned by FIMO are considered. Therefore, the candidate binding sites which does not match the prediction for each method were considered negative.

## Results and discussion

### Transcription factors (TFs) show different chromatin patterns surrounding their binding sites

**Fig. 1:**
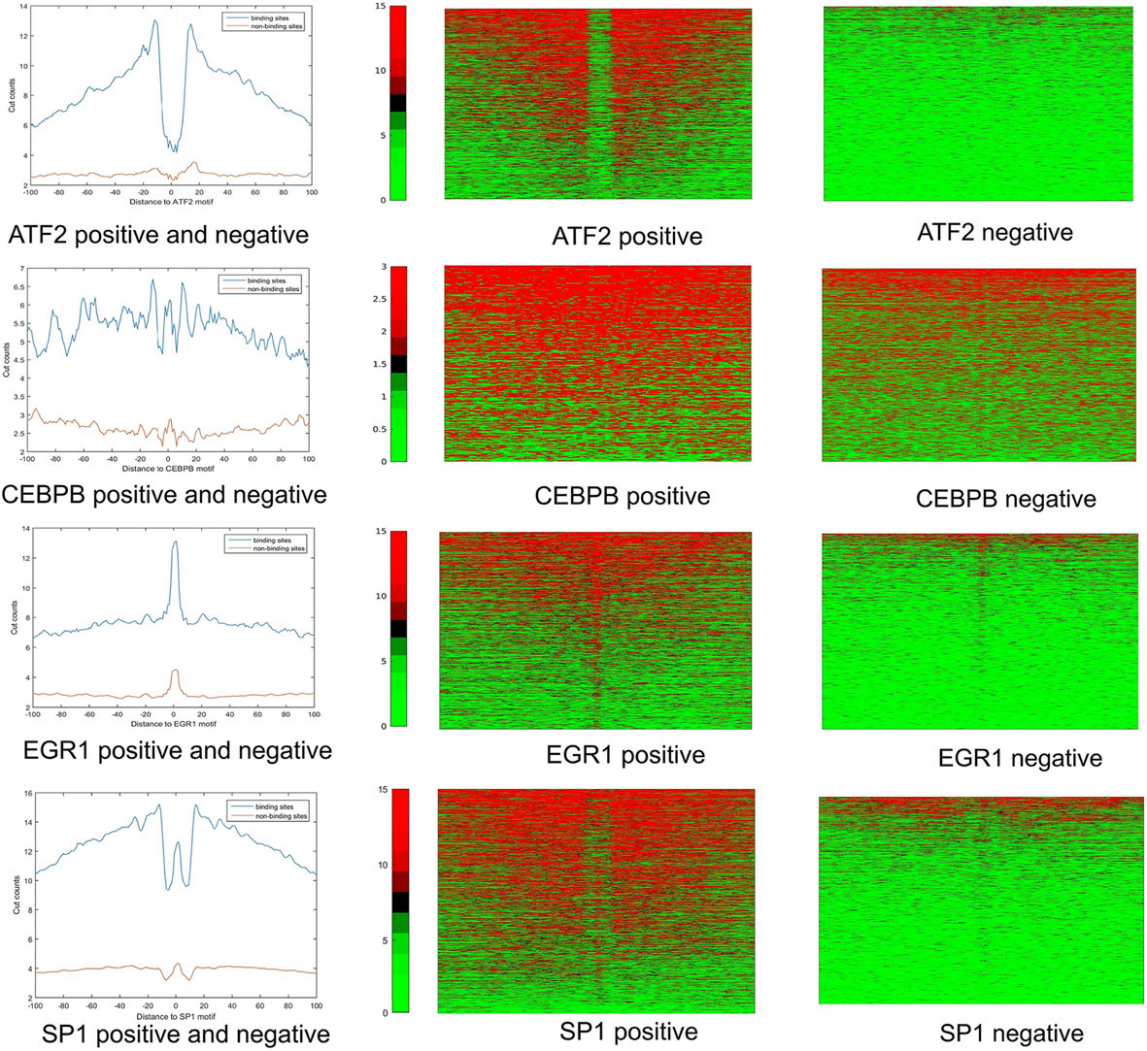
Cut profiles around motif sites show different patterns. The *left* panel shows the average cut counts around binding sites for bounded sites (positive) and unbounded sites (negative) respectively. The *right* panel shows cut counts for each individual site from positive set

We first assessed the patterns of TF footprints using DNase-Seq profiles. Detailed analysis of individual motif sites for the TFs revealed complex footprints structures. For this purpose, we integrated TF ChIP-Seq and DNase-Seq profiles, and analyzed the DNase-Seq profiles surrounding the TF binding sites identified by ChIP-Seq. The positions of ChIP-Seq peaks formed a positive set. In the meantime, we also searched for the presence of TF binding motifs within the DNase-Seq regions. Sites with the matched motif outside the ChIP-Seq peaks were considered as negatives. As shown in Fig. 1, the DNase-Seq profiles were shown for a few representative TFs. ATF2 illustrates a typical footprint structure. Most of ATF2 binding sites determined by ChIP-Seq have low DNase-Seq cut profiles and high cut profiles at the flanking regions. For comparison, we examined the DNase-Seq profiles around the negative set for ATF2. The overall cut profiles are much lower surrounding these sites, suggesting that cut profiles (or peak intensity) of DNase-Seq profiles are one major determinant for the ATF2 binding events.

In contrast, however, other factors such as CEBPB, ERG1 and SP1 did not show obvious footprints surrounding their binding sites. For example, the cut profiles at the center of CEBPB binding sites are almost similar to those in the flanking regions. Interestingly, although the average DNase-Seq intensities at the sites from the negative set are lower than those from the positive set, many sites from the negative set also have high cut profiles, suggesting that cut profiles obtained from DNase-Seq profiles are not good predictors for CEBPB binding events.

The cut profiles for ERG1 showed an “inverse” footprint pattern, in that the cut profiles are much higher at the center of ERG1 binding sites than in the flanking regions. A similar pattern was observed for the negative set. In addition, SP1 showed a more complex footprint pattern, combining regular footprint and “inverse” footprint patterns. Bias corrected [27] did not change the overall patterns for these factors.

Our analyses suggested that a footprint-based approach might not be effective to identifying TF binding sites due to the complex nature of footprints. Approaches solely based on the DNase-Seq profiles cannot best separate the true binding sites and the sites in the negative set. For example, many sites in CEBPB negative set have comparable cut profiles to the real CEBPB binding sites. This analysis suggests that TFs have different chromatin accessibility patterns surrounding their binding sites. It raises the question whether we could have a universal computational model or we need TF-specific models for different TFs.

### Evaluate the transferability of prediction across different TFs and cell types

**Fig. 2:**
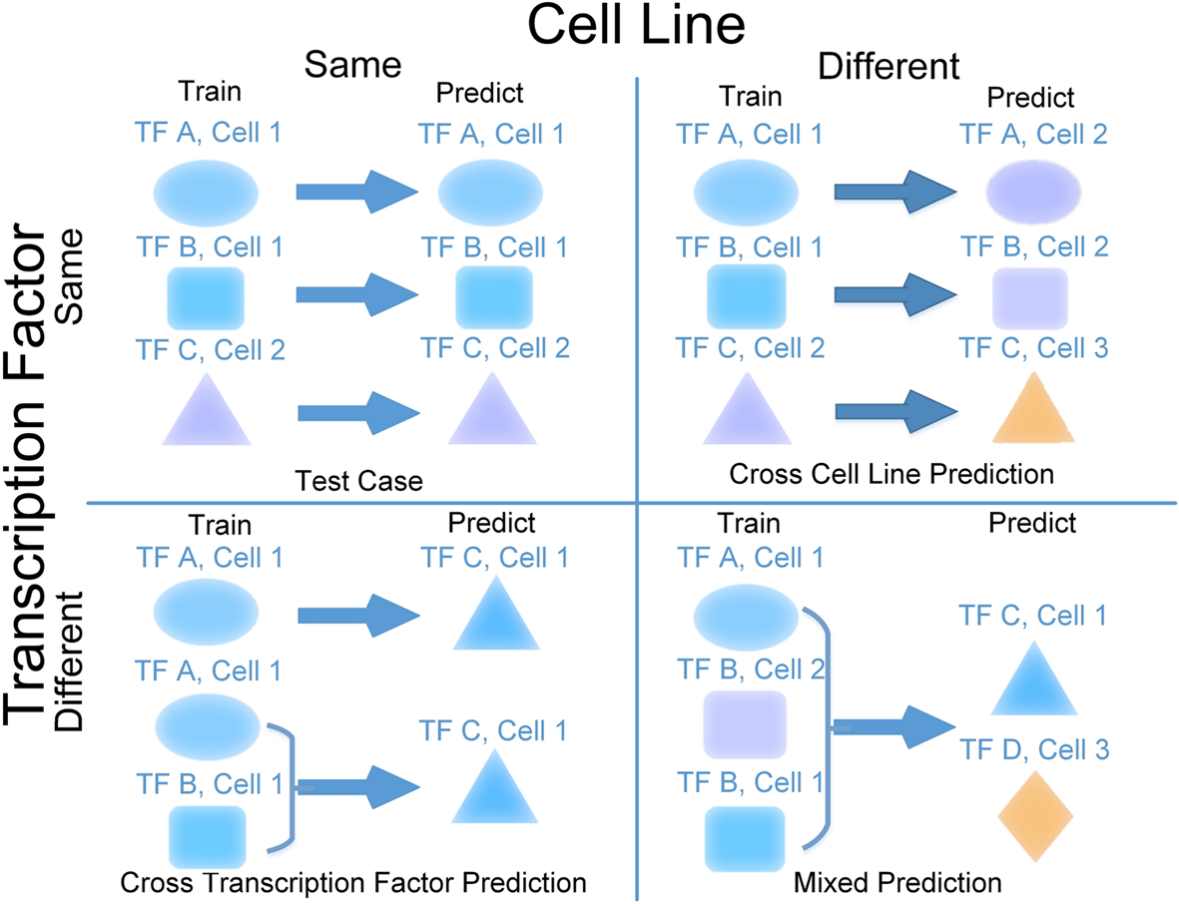
Different scenarios of prediction using ChIP-Seq as ground truth

We first described the problem setting for our prediction of TF binding sites (Fig. 2). Two most basic requirements for the prediction are (1) the binding motif of a particular TF, which is often represented by a PWM, and (2) the chromatin accessibility data (DNase-Seq or ATAC-Seq) for a cell type of interest. We first scan the motif within the chromatin accessible regions and obtain a set of matched positions in these regions. We then attempt to determine the true TFBS among these matched positions. Our prediction is a supervised learning approach, which is based on the ChIP-Seq data showing the genome-wide binding sites for a given TF. We have four scenarios based on available ChIP-Seq datasets.

(1) The ChIP-Seq data of the TF in the cell type of interest is available. In practice, we do not need to predict the binding sites of TF because the ChIP-Seq data already provide the binding events of the TF. However, we could train a model using 2/3 of all binding sites, and use this to predict the binding sites for the remaining 1/3 of all binding sites. The prediction serves as a benchmark and was used to test the performance of the model. We termed this type of prediction as self-prediction.

(2) The ChIP-Seq data of other TFs in the cell type of interest are available. We train a model to use the other TF and use the model to predict the binding site of the TF of interest. In addition, we can combine ChIP-Seq data for multiple TFs for training and predicted the binding sites for the TF (Fig. 2). We termed this type of prediction as cross-TFs prediction (Fig. 2).

(3) The ChIP-Seq data for the TF of interest in other cell type is available. In this situation, we also require the chromatin accessibility data for that cell type. We will train the model in other cell type, and predict the binding sites of the TF in the cell type of interest. We termed this type of prediction as cross-cell type prediction (Fig. 2).

(4) The ChIP-Seq data for other TF in other cell type are available. In this situation too, we require the chromatin accessibility data in the other cell type. We termed this prediction as mixed prediction (Fig. 2).

### Self-prediction: combination of static and dynamic features increases prediction performance

**Fig. 3:**
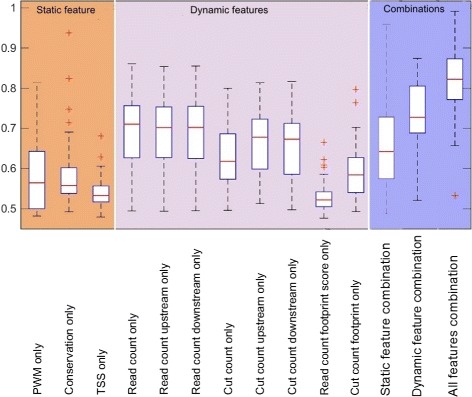
Combination of static and dynamic features increases prediction performance. Boxplot of AUC of 34 different TF motifs using selected features

Our algorithm, BPAC (TF Binding Prediction from accessibility data), used a random forest model to predict the TF binding sites in a cell type with available chromatin accessibility information such as DNase-Seq or ATAC-Seq. We first identify the features that can be used for the prediction. The features belong to mainly two categories – static and dynamic. Static features include PWM score, evolutionary conservation score, and distance to TSS. For a given TF, these features do not change with respect to different cell types. Dynamic features are derived from chromatin accessibility data, including read profiles at, upstream, and downstream from candidate TFBS, cut profiles at, upstream, and downstream from candidate TFBS, footprint scores obtained from read profiles and cut profiles. These features are cell type specific. For a given TF, we used 2/3 of binding sites identified by ChIP-Seq for training and evaluated the prediction using the remaining 1/3 of binding sites. A random forest model was trained and then used to make the prediction. The performance was measured by AUC and AUPR. We first evaluated different features using 34 TF ChIP-Seq datasets obtained from GM12878 cells. As shown in Fig. 3, for the static features, the AUC ranges from 0.5 to 0.62 using individual feature alone (0.17 to 0.24 for AUPR). PWM score achieved the highest average among three static features, with the average AUC of 0.55, average AUPR of 0.23. This finding confirms that sequence specificity of TFs plays an important role in TF binding events. We also noticed that the AUC and AUPR for PWM showed a large variance, indicating that the binding motifs for some TFs have substantially better prediction power than others.

Among the dynamic features, the read profile at the motif sequence and its flanking regions (upstream and downstream) present the highest performance (AUC=0.70, AUPR=0.28). This is higher than cut profile footprint score (AUC=0.58, AUPR=0.21). In this sense, read profiles alone can provide high prediction performance. However, the read profile footprint score, which combines the read profiles at the center and flanking regions of candidate binding sites, is not informative in identifying TF binding (AUC=0.52, AUPR=0.16).

Combining all static features improves prediction accuracy, with average AUC of 0.65 (AUPR=0.23). The combination of dynamic features improves prediction accuracy relative to comparing single dynamic features. The prediction achieved the highest performance when a combination of all static features and dynamic features was analyzed, with the average AUC reached to 0.81 and average AUPR reached to 0.37. In the following analysis, we used the combination of static and dynamic features.

### Cross-TF prediction is comparable with self-prediction

We then evaluated whether the ChIP-Seq data for other TFs can be used to predict the binding events for the TF of interest. For this purpose, we obtained 23 TF ChIP-Seq in GM12878 cell line. We trained a random forest model based on each TF and used the model to predict the binding sites for every individual TF, including the TF for training. The performance of self-prediction ranged from 0.71 to 0.92 for AUC, 0.01 to 0.87 for AUPR. Interestingly, majority of the cross-TF predictions based on other TFs achieved the similar performance with an overall mean AUC of 0.77, mean AUPR of 0.36.

**Fig. 4:**
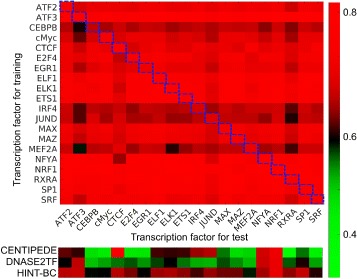
Performance of cross-TF predictions. The TFs shown in Y-axis were used for training and the binding sites of TFs shown in X-axis were predicted. The cells highlighted in *blue* boxes are the self-prediction, which were used as a benchmark. The models were constructed from a TF motif in GM12878. The color showed the AUC for each prediction. The *bottom* panel shows the results using CENTIPEDE, DNASE2TFand HINT-BC for these TFs

**Fig. 5:**
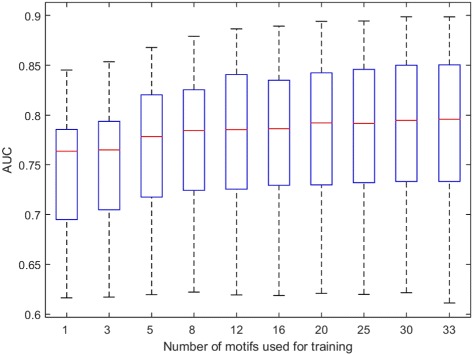
Average AUC increases with number of training motifs. As the number of motifs used for training increases, the average AUC of prediction of all motifs increases

However, individual TFs showed substantially different prediction performances (Fig. 4). Some TFs (e.g., ATF3, RXRA, NRF1) generate models (good predictor TFs) that predict the binding events for other TFs well, while other TFs (e.g. CEBPB, IRF4, JUND, MEF2A) generate models (poor predictor TFs) with less satisfactory performance. On the other hand, some TFs (e.g. EGR1, ELF1) have higher prediction performance than most of the training models used (properly predicted TFs), while other TFs (e.g., ATF3, JUND, RXRA) have lower prediction performance than most of training models used (poorly predicted TFs). We found that correlation between a TFs’ prediction performance and its binding motif’s information content is very weak (0.29). The result suggests that the sequence motif is not a major determinant for properly or poorly predicted TFs. In practice, we can choose good predictor TFs as models to predict the target TF’s binding (Fig. 4). For example, although JUND is a poorly predicted TF, from Fig. 5 we see that NRF1 is a good predictor TF for JUND. We can thus use a model constructed from NRF1 to predict the location of JUND binding sites.

We also compare our approach with three representative methods: CENTIPEDE [29], DNASE2TF [24] and HINT-BC [26]. The former is an unsupervised learning approach, and the latter two identify footprints of TF binding. Our approach outperformed these methods with the dataset. Specifically, HINT-BC and CENTIPEDE achieves better prediction than DNASE2TF (Fig. 4). This agrees with results from Sung et al. [24].

### Models obtained from multiple TFs are better than those generated using a single TF

We further studied whether increasing the number of TF motifs used for training increases the accuracy of TFBS prediction. For each N (*N*=3, 5, 8, 12, 16, 20, 25, 30), we randomly chose 100 combinations of N TFs. For each combination, data from these N TF motifs are used for model training. The model is then used to predict the binding sites for a target TF, which is not included in the N TFs. It is clear that the average performance for the prediction of target TF binding sites increases with the number of TF motifs used for training (Fig. 5). When the number of motifs used for training is 30, there is a significant difference in predictivity comparing with those training with only one motif (*p* =0.0086).

**Fig. 6:**
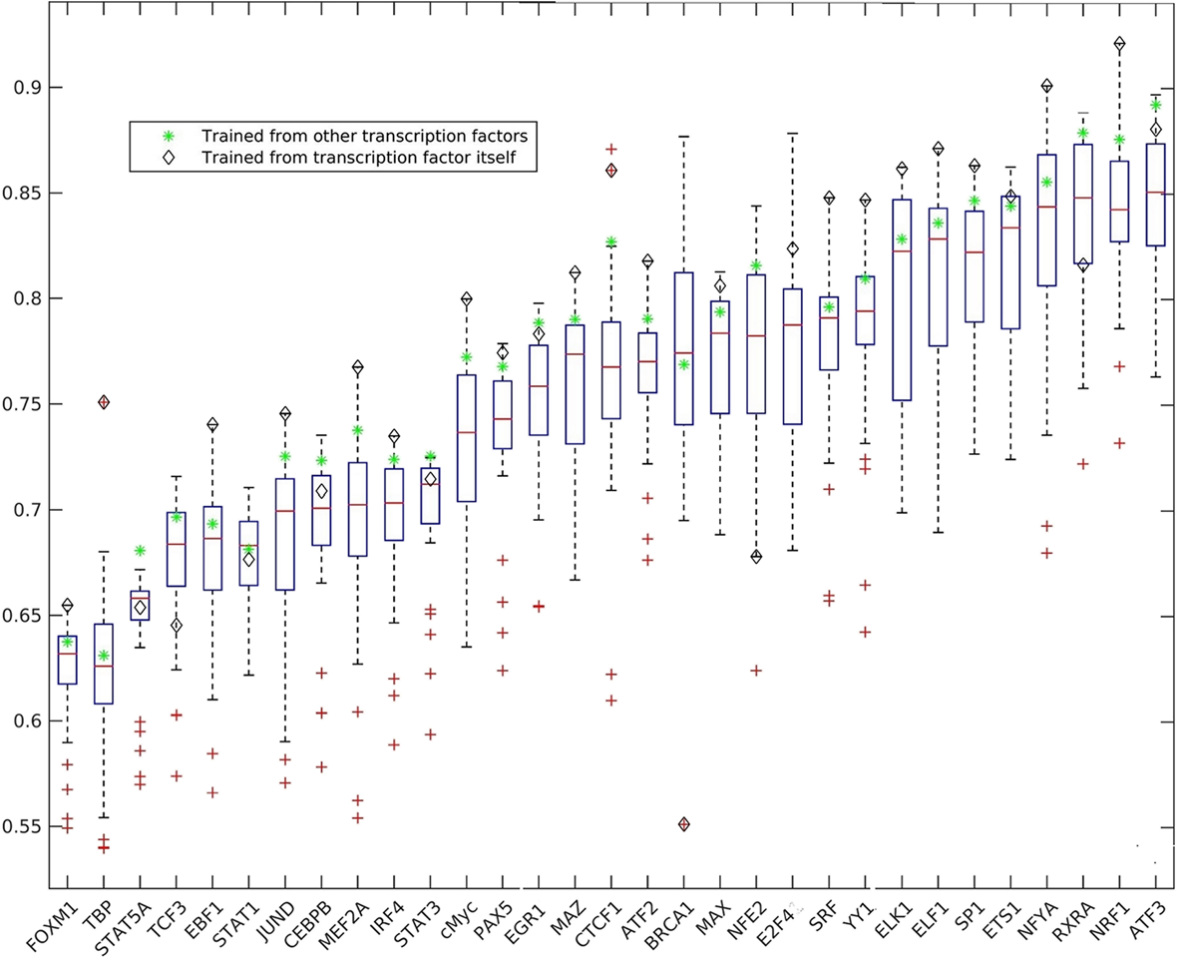
Combination of mutliple TF motifs. Prediction combining the profiles of multiple TF motifs is significantly better than prediction using the profile of a single TF motif. Boxplot is cross-TF prediction using single TF for training. *Green* asterisks denote the cross-TF prediction multiple TF motifs for training. *Diamonds* are the self-prediction

As a benchmark, we also predicted the binding sites of a TF using self-prediction (i.e. 2/3 of the binding sites for training, and the remaining 1/3 for prediction). We compared the performance for 31 TFs in GM12878. We performed cross-TF prediction using 30 TFs for training. In most cases, models based on the 30 TFs performed better than models based on single TFs (Fig. 6). Furthermore, the model based on 30 TFs achieved almost the same performance as the self-prediction model (Fig. 6). Taken together, our study suggested that a model based on multiple TFs is a more reliable tool for predicting the binding sites for a novel TF.

### Cross-cell line prediction is comparable with self-prediction

We next studied a situation where we have the ChIP-Seq for a TF in one cell line, and sought to predict its binding sites in another cell line, in a case where both cell lines have data for chromatin accessibility. For example, if we trained a random forest model of ATF3 in GM12878 cell and predicted its binding sites in A549, H1-hESC, and K562 cells, we obtained the AUC of 0.89, 0.80, and 0.81, respectively. As a benchmark, the AUC of ATF3 self-prediction in GM12878 cell is 0.87, suggesting that we could transfer the model learned from one cell type to a different cell line.

**Fig. 7:**
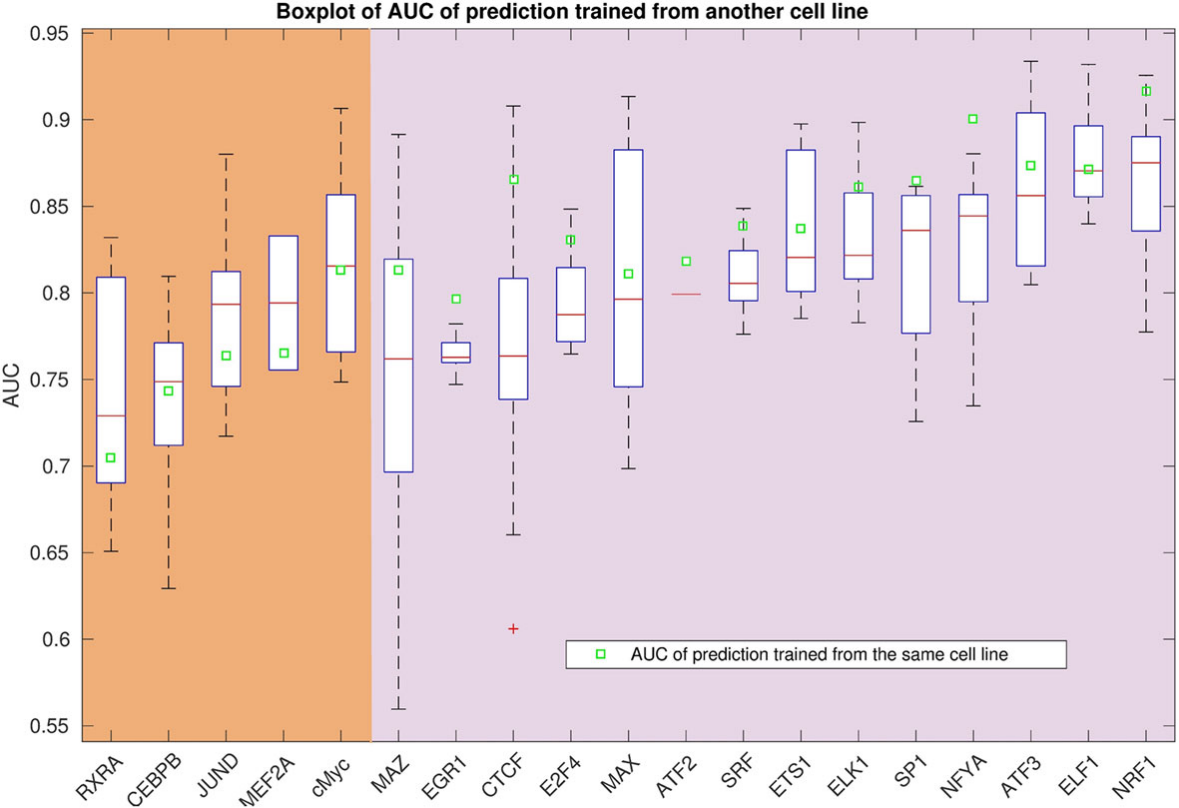
Result on cross-cell type prediction. Cross-cell prediction for 19 TFs. As comparison, the performance of the self-prediction was indicated by *green* square

Figure 7 summarizes the performance of cross-cell prediction for 19 TFs. These TFs have ChIP-Seq obtained from multiple different cell types, along with chromatin accessibility data for the corresponding cell types. For each TF, we learned the models from one cell type and predicted the binding events in other cell types. We have total 3-20 cross-cell prediction for each TF. For comparison, we also indicated the performance of self-prediction in these cell types as benchmark (green squares in Fig. 7). Among 19 TFs, 14 showed that self-prediction performs better than the average performance of cross-cell prediction. Interestingly, five TFs have better cross-cell prediction for most of cell types than for self-prediction (panel with brown background in Fig. 7). These factors are either poor predictor TFs or poorly predicted TFs. This suggests that using information from additional cell lines can help improve the TFBS prediction for some poorly predicted TFs.

### Mixed prediction is also comparable with self-prediction

**Fig. 8:**
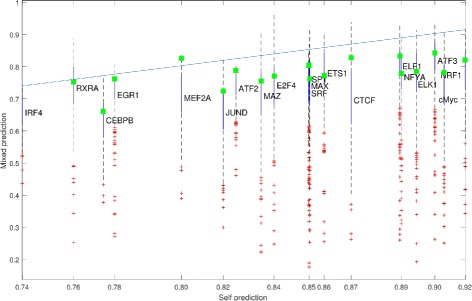
Mixed prediction is also comparable with prediction using profiles of self-transcription factor. 100 random repeats using data from single TF motif for training regardless cell line were made for each target TF motif. *Green* Square is result of single TF motif binding prediction from model constructed from 34 TFs together

We then examined the performance of the mixed prediction, in which we learned the model from other TFs in other cell types. When we performed 8855 cross prediction analyses for 20 TFs in six cell types, the corresponding average AUC ranged from 0.63 to 0.82. We compared the performance of mixed prediction with self-prediction, and found that for most TFs, mixed prediction performed less well than self-prediction (Fig. 8). Nevertheless, the performance of mixed prediction is still acceptable in terms of AUC. The above results suggested that we could build a universal model using existing ChIP-Seq data from many TFs in multiple cell types. This universal model can then be used to predict the TF binding sites in any cell type, so long as the chromatin accessibility data are available for the cell type of interest. We developed a program, BPAC (TF Binding Prediction from ACcessibility data), and made it available as an online web tool.

The source code and documentation are freely available under the GNU General Public License via GitHub at http://github.com/sliu2/BPAC. A web server is also available at http://bioinfo.wilmer.jhu.edu/BPAC. As shown in the website, user can provide different type of inputs according to different situations. If TF motif is not given, we use STAMP tools [46, 47] to get most probable motif.

## Conclusions

In this work, we proposed a supervised classification approach to predict TF binding events, using available TF ChIP-Seq data as a gold standard. The features are selected from sequence related information, gene related information, and chromatin accessibility information. There are cases that based on sequence information, or gene related information, or chromatin accessibility information alone, some TFs have poor predictivity because of limitation of each type of information. We show that combining these information improves the prediction. One key question related to the general usefulness of this approach is whether or not the model learned from other TFs in other cell types is transferable. We assessed the transferability for many TFs and different cell lines, and discovered that in most cases a model learning from other TFs, especially the combination of many TFs, performed almost as well as the model learned from the target TF. The analysis suggested that we could build a universal model for prediction of TF binding sites. However, we would like to emphasize that the focus of this paper is to access the model transferability across TFs and cell lines, rather than developing the most powerful model for TF binding prediction. We believe that some genomic features such as cofactor PWMs are important to improve the prediction. However, these features might not be suitable for our purpose because they may not be transferable across different cell lines. For example, different cofactors might co-exist with one TF in different cell lines. Therefore, we used a basic model with small number of features to assess the model transferability. Based on the analysis of human TFs, it seems that the model can be used to predict on any TFs, on any cell type, provided that the TF binding motif (i.e. PWM) and the chromatin accessibility of the target cell type are known. Of course, the transferability across species requires further investigation. Previous analysis has shown that some TFs like CTCF are transferable cross cell lines without loss of predictability [34], our study provided a more comprehensive assessment of the model transferability for much more TFs and cell types.

## Additional file

Additional file 1 Supplementary information about data used in this study. This file contains the following tables: **Table S1** – Transcription factor motifs used in this study. **Table S2** – Dnase-Seq (bam format) and ChIP-Seq (narrowPeak format) used in this study. (PDF 23 kb)

## Abbreviations

AUC: Area under receiver operation characteristic
AUPR: Area under precision recall
FN: False negative
FP: False positive
FPR: False positive rate
PWM: Position weight matrix
ROC: Receiver operating characteristic
SVM: Support vector machine
TF: Transcription factor
TFBS: Transcription factor binding sites
TN: True negative
TP: True positive
TPR: True positive rate
TSS: Transcription start sites
