# Supplementary material for: Assessing the model transferability for prediction of transcription factor binding sites based on chromatin accessibility
Source: BMC Bioinformatics. 2017 Jul 27;18:355. doi: 10.1186/s12859-017-1769-7 (PMC5530957; doi:10.1186/s12859-017-1769-7)
Supplement: Additional file 1 — Supplementary information about data used in this study. This file contains the following tables: Table S1 – Transcription factor motifs used in this study. Table S2 – Dnase-Seq (bam format) and ChIP-Seq (narrowPeak format) used in this study. (PDF 23 kb) [file 12859_2017_1769_MOESM1_ESM.pdf]

Supplementary tables

Table S1 Transcription factor (TF) motifs used in the article**.**

TF	TRANSFAC Motifs	
ATF2	M01862	
ATF3	M00513	
BRCA1	M01082	
CEBPB	M01896	
cMyc	M01145	
CTCF	M01200	M01259
E2F4	M00738	M00739
EBF1	M02267	
EGR1	M02848	M02744
ELF1	M00110	
ELK1	M00007	
ETS1	M00339	
FOXM1	M00630	
IRF4	M02768	
JUND	M03552	
MAX	M02881	
MAZ	M00649	
MEF2A	M01301	
NFE2	M00037	
NFYA	M02106	
NRF1	M00652	
PAX5	M03577	
RXRA	M02791	
SP1	M00008	
SRF	M01257	
STAT1	M01260	
STAT3	M01595	
STAT5A	M01890	
TBP	M02814	
TCF3	M02816	
YY1	M00793	

Table S2 Dnase-Seq and ChIP-seq used in the article**.**

**Cell line**	**Dnase-Seq**	**Used in Figures**	**ChIP-Seq**
GM12878	ENCFF000SKV<br>ENCFF000SKZ<br>ENCFF000SLD<br>ENCFF000SKW<br>ENCFF000SLB	1,3,4,5,6,7,8	http://hgdownload.soe.ucsc.edu/goldenPath/hg19/encodeDCC/wgEncodeAwgTfbsUniform/
K562	ENCFF000SVI<br>ENCFF000SVN<br>ENCFF000SVO	7,8	
H1-ESC	ENCFF000SOA<br>ENCFF000SOC	7,8	
Hela-S3	ENCFF001DCR<br>ENCFF001DCQ	7,8	
HepG2	ENCFF001BDJ	7,8	
A549	ENCFF001ARO	7,8	
